# A Potent Chemotherapeutic Strategy with Eg5 Inhibitor against Gemcitabine Resistant Bladder Cancer

**DOI:** 10.1371/journal.pone.0144484

**Published:** 2015-12-10

**Authors:** Liang Sun, Jiaju Lu, Zhihong Niu, Kejia Ding, Dongbin Bi, Shuai Liu, Jiamei Li, Fei Wu, Hui Zhang, Zuohui Zhao, Sentai Ding

**Affiliations:** 1 Department of Urology, Shandong Provincial Hospital Affiliated to Shandong University, Jinan, ShanDong, China; 2 Department of pathology, Shandong Provincial Hospital Affiliated to Shandong University, Jinan, ShanDong, China; 3 Department of Cardiac Surgery, The Second Affiliated Hospital of Shandong University of Traditional Chinese Medicine, Jinan, ShanDong, China; Columbia University, UNITED STATES

## Abstract

Development of resistance to gemcitabine is a major concern in bladder cancer therapy, and the mechanism remains unclear. Eg5 has been recently identified as an attractive target in cancer chemotherapy, so novel targeted chemotherapy with Eg5 inhibitor is expected to improve the anticancer effect in gemcitabine-resistant bladder cancer. In this research, RT112-Gr cells were 350-fold less sensitive to gemcitabine than the parental cell lines, while KU7-Gr cells were 15-fold less sensitive to gemcitabine than the parental cell lines. Human OneArray Microarray analysis was performed to obtain broad spectrum information about the genes differentially expressed in RT112 and RT112-Gr cells. The anti-proliferative activity of S(MeO)TLC, an Eg5 inhibitor, was analyzed in RT112-Gr cell lines using a cell viability assay. Furthermore, the inhibitory effect was evaluated in vivo using subcutaneous xenograft tumor model. According to the result of Human OneArray® GeneChip, RRM1 and RRM2 were up-regulated, while there was no significant change in Eg5. Trypan blue staining confirmed that in S(MeO)TLC and Gemcitabine combining S(MeO)TLC group cell viability were significantly decreased in RT112-Gr cells as compared with other groups. S(MeO)TLC and S(MeO)TLC+gemcitabine groups prominently suppressed tumor growth in comparison with other groups’ in vivo. There were no significant differences in S(MeO)TLC and gemcitabine+S(MeO)TLC group in the effect of inhibition of bladder cancer in vivo and in vitro. Our data collectively demonstrated that S(MeO)TLC represents a novel strategy for the treatment of gemcitabine resistant bladder cancer.

## Introduction

Bladder cancer (BCa) represents the fourth most common cancer in the United States[1,2]. Approximately 25% of bladder cancer patients are diagnosed with muscle-invasive bladder cancer (MIBC), although 75% of newly diagnosed tumors are non–muscle invasive (Ta, Tis, and T1); most of them recur and 15–20% progress to invade tunica muscularis. And the vast majority of cancer-specific deaths are due to MIBC, leading to local invasion and distant metastasis [3, 4]. The mortality of the disease urges urologists to explore novel methods to treat bladder cancer[5]. Chemotherapy with gemcitabine and cisplatin is the most popular option for bladder cancer. Gemcitabine is an analog of deoxycytidine with high activity against many types of solid tumors including pancreatic, cervical, ovarian, breast, bladder, and non-small cell lung cancers[6,7]. However, the development of resistance to gemcitabine is now a major concern to urologists. Despite a reasonable response rate after initial chemotherapy in patients with metastatic bladder cancer, 60–70% of responding patients relapse within the first year, with a median survival of 12–14 months. This limited efficacy may be due to de novo drug resistance and the development of cellular drug-resistant phenotype during treatment[8].

However, the underlying mechanisms of inducing chemotherapy resistance by Gemcitabine remain unknown. Recently, through the study of pancreatic cancer, Nakahira S et al reported an important factor in gemcitabine resistance was the overexpression of ribonucleotide reductase (RR)[9]. RR consists of the dimerized large and small subunits, M1 and M2, respectively. The M1 subunit possesses a binding site for enzyme regulation (regulatory subunit), and the M2 subunit is involved with RR activity (catalytic subunit)[10]. RRM1 is supposed to play a role in gemcitabine resistance of the variety of cancer as metabolic enzymes of the drug[9, 11]. RRM1 is not only a cellular target for gemcitabine, but also a tumor suppressor. Preclinical studies have demonstrated its involvement in the suppression of cancer cell proliferation, migration, and metastasis[12, 13]. In some cancers, a high level of RRM2 mRNA correlates with chemotherapeutic resistance, cellular invasiveness and unsatisfied prognosis, suggesting that RRM2 contributes to malignant progression and is a potential therapeutic target. However, there is limited information concerning RRM1 and RRM2 protein expression in bladder cancer, and to our knowledge no reports exist describing the role of RRM in the process of drug resistance in bladder cancer. Moreover, some recent studies have indicated that RRM plays an important role in the development and progression of human carcinomas, but the clinical significance of RRM expression in BCa remains unclear.

On the other hand, it is of great significance to investigate novel bladder cancer chemotherapeutic strategy. Targeted drugs in the treatment of urinary tract tumors in recent years showed promising results. Our early studies have found that Eg5 inhibitors as targeted drugs in vivo and in vitro treatment of prostate cancer and bladder cancer should have satisfying curative effects[14, 15]. Eg5, a key molecule involved in the formation of bipolar spindles, is one of the most attractive target enzymes in antimitotic drug discovery [16]. Eg5 accounts for many of the movements of the spindle and chromosomes in dividing cells and localizes to the spindle in mitotic dividing cells[17]. An interesting feature of Eg5 is that it localizes to microtubules in mitosis, but not to interphase microtubules, suggesting that an Eg5 inhibitor may be useful to specifically target proliferating tumor tissue[18]. Several chemical types of small molecule Eg5 inhibitors have been reported[16]. S-(4-methoxytrityl)-L-cysteine (S(MeO)TLC), a derivative of S-trityl- L-cysteine (STLC), can specifically inhibit Eg5, and induce monopolar mitotic spindle formation[14, 15]. Failure of Eg5 function leads to cell cycle arrest in mitosis with monoastral microtubule arrays. The important role of Eg5 in mitotic progression makes it an attractive candidate for developing targeted therapy in cancer[19]. However, there is no study of Eg5 inhibitor treatment of Gemcitabine resistant bladder cancer.

In this study, a gemcitabine-resistant human bladder cancer cell line was established, and the role of RRM1 and RRM2 in the development of gemcitabine resistance was initially investigated. We also assessed the efficacy of the anticancer effect of Eg5 targeted therapy in vitro and in vivo, aiming to offer a better clinical treatment strategy for patients with gemcitabine resistant bladder cancer.

## Materials and Methods

### Ethics Statement

This study was carried out in strict accordance with the recommendations in the Guide for the Care and Use of Laboratory Animals of the National Institutes of Health. The protocol was approved by the Committee on the Ethics of Animal Experiments of Provincial Hospital Affiliated to Shandong University (Permit Number: 2013–026). All surgery was performed under sodium pentobarbital anesthesia, and all efforts were made to minimize suffering. Bladder cancer tissues were collected from Shandong Provincial Hospital Affiliated to Shandong University. Before the study, the protocol was approved by the Ethics Committee of Provincial Hospital Affiliated to Shandong University (Permit Number: 2012–033). Written informed consents were obtained from all patients.

### Cells, reagents and clinical samples

Bladder cancer cell line RT112 and KU7 were used in this study and cultured as described previously[14,20]. RT112 and KU7 cell lines were purchased from cell bank of Chinese Academy of Science (Shanghai, China). RT112 and KU7 cells were mainly used in the following study, which originate from non-invasive bladder cancer. The cells were cultured in RPMI-1640 supplemented with 10% fetal bovine serum and 1% penicillin/streptomycin, and incubated at 37°C with a humidified 5% CO^2^ atmosphere.

The Eg5 inhibitors, S(MeO)TLC were purchased from Bachem (Bubendorf, Switzerland) and were dissolved in dimethyl sulfoxide (DMSO). Antibody anti-RRM1 and anti-RRM2 (Rabbit) was purchased from Abcam (USA); IRDye 800CW conjugated goat anti-rabbit IgG were from Li-Cor Biosciences (USA). Prime Script RT-PCR kit was purchased from TaKaRa (China). TRIzol was purchased from Invitrogen (USA).

140 cases of bladder cancer tissues were collected from Shandong Provincial Hospital Affiliated to Shandong University from December 2005 to March 2007. Radiotherapy, chemotherapy and immunotherapy were not performed before the surgery and all samples were verified by two pathologies after the surgery.

### Establishment of the gemcitabine-resistant cell lines

Gemcitabine-resistant cells were developed by chronic, repeated exposure to gemcitabine though gradient culture. The established resistant cell line was maintained in medium containing 45 nmol/L of gemcitabine. For all studies, resistant cells were cultured in drug-free medium for 1 week to eliminate gemcitabine. Gemcitabine-resistant cells are referred as RT112-Gr cells and KU7-Gr. IC50 of Gemcitabine-resistant RT112 cells to Gemcitabine is 4.2umol/L. IC50 of Gemcitabine-resistant KU7 cells to Gemcitabine is 0.285umol/L. So we choose RT112-Gr cells as a subject in the following experiments.

### Human OneArray Microarray

Total RNA was extracted by Trizol reagent (Invitrogen, Shanghai, CN) and sent to the Phalanx Biotech Group (Shanghai, CN) for expression microarray analysis. Triplicate hybridizations for each sample were performed using the Human Whole-Genome OneArray 6.1.

### RRM1 and RRM2 expression analysis by IHC

Immunohistochemical analysis was performed to assay RRM1 and RRM2 expression in bladder cancer, as described previously[21]. Anti-RRM1 and Anti-RRM2 antibodies were both used at a dilution of 1:100. Negative controls consisted of slides where the primary antibody had been omitted. Cases were evaluated as having positive staining if >10% of the cytoplasm of the tumor cells was stained. Tumor grade and stage were evaluated using standard hematoxylin and eosin (H&E) staining.

### Inhibition of RRM1/2 expression by RNA interference

RT112-Gr Cells (2x10^5^) were seeded in 6-well plates in triplicates and after 12h incubation, the cells were transfected with various concentrations of siRNA using HiPerfect Reagent (Qiagen) in accord to the manufacturer's instruction. The small interference RNA used to target RRM1/2 mRNA sequence was synthesized by Qiagen.

### Cell viability assay

Cell viability following treatment with Eg5 inhibitors was assessed using the 3-(4, 5-dimethylthiazol-2-yl)-2, 5-diphenyltetrazolium bromide (MTT) assay, as described previously[20]. Cells (2 × 10^3^) were seeded in 96-well plates and incubated for 24 h. After that, gemcitabine, S(MeO)TLC, gemcitabine+S(MeO)TLC and vehicle (DMSO) were added at the indicated concentration in triplicate wells and cell viability was measured after 24 h, 48 h and 72 h of treatment, respectively. The concentrations of 50% cell growth inhibition (IC50) were calculated according to SPSS17.0 Software.

### Hoechst staining

After administration with 45nM gemcitabine, 0.5μM S(MeO)TLC, 45nM gemcitabine+0.5μM S(MeO) TLC or vehicle for 24 h, 48h and 72 h, RT112-Gr cells were stained with 1 mM Hoechst 33342 solution (Santa Cruz, Dallas, USA) for nuclear staining and analyzed with a fluorescence microscope. The cells with typical condensation and fragmentation of the nuclei were visualized and identified as apoptotic cells.

### Reverse Transcription-PCR analysis

RRM1 and RRM2 expressions in bladder cancer cell lines were determined by RT-PCR analysis using standard methods as described previously[22]. Total RNA was extracted from RT112 and RT112-Gr cell pellets using the TRIzol reagent and amplified by reverse transcription-PCR (RT-PCR) according to the manufacturer’s instructions, respectively. A housekeeping gene, glyceraldehydes phosphate dehydrogenase (GAPDH), was used as an endogenous control. GAPDH and RRM1 and RRM2 primers were constructed by using Primer 5 Software and verified by DNA sequencing analysis. GAPDH (1382 bp) primers were: Forward:5´-GATGCTGCGCCTGCGGTAGA-3´, Reverse: 5´-TTGGTTGAGCACAGGGTAC-3´; RRM1(391bp) primers were: Forward:5´-GTTGTATTCGGGCTACTGG-3´, Reverse:5´-ACTTTGCGGACACGACCT-3´; RRM2(882bp) primers were: Forward:5´-GCCGCTTTGTCATCTTCC-3´, Reverse: 5´-TCCTCTGATACTCGCCTACT-3´. PCR products were then electrophoresed in 1.0% agarose gel and visualized by a transilluminator. The pixel intensity for each band was determined using the AlphaImager^TM^2200.

### Animal experiment

Six to eight weeks old female BALB/c nude mice were purchased from Vital River Company (Beijing, China). The study was obtained approval from the Committee on Animal Research of Shandong Provincial Hospital of Shandong University and our care was in accordance with institution guidelines.

For subcutaneous xenograft models, approximately 4×10^6^ RT112-Gr cells (suspended in 100μl PBS) were inoculated subcutaneously into both flanks per mouse, as described previously [23]. The tumor volume was calculated as follows: volume = Width^2^× Length/2. When tumors were palpable and measurable (about 50mm^3^), 20 mice were randomly divided into four groups and treated once daily (five days a week) by intraperitoneal injection with DMSO (vehicle control), 20 mg/kg S(MeO)TLC, 50 mg/kg gemcitabine or 20mg/kg S(MeO)TLC +50mg/kg gemcitabine for 5 days. Mice body weighs and tumor sizes were measured every other day. The other mice were sacrificed on the 21st day after the initial treatment, and tumors were also excised and paraffin-embedded for H&E staining.

### Statistical analysis

The significance of the relationships between RRM1 and RRM2 expression and clinicopathological parameters was evaluated using chi-squared tests and two-way ANOVA. Means were compared using a two-tailed student's *t* test and the inhibitory activity of S(MeO)TLC and gemcitabine on xenograft models were performed with a two-way ANOVA followed by S-N-K test. SPSS 17.0 software was used as statistical analysis. *P* < 0.05 was considered as statistically significant.

## Results

### Global gene expression analysis in RT112 and RT112-Gr cell lines

RT112-Gr cells were 350-fold less sensitive to gemcitabine than the parental cell lines, which IC50 was 4.2 umol/l in former. KU7-Gr cells were 15-fold less sensitive to gemcitabine than the parental cell lines, which IC50 was 0.285umol/l in former. Microarray analysis was performed to obtain broad spectrum information about the genes differentially expressed in RT112 and RT112-Gr cells. The RNAs obtained from RT112 and RT112-Gr cells were amplified, fragmented, labeled, and hybridized to GeneChip microarrays. Of the 55752 genes represented on the Human OneArray® GeneChip, 442 showed significantly up-regulated and 235 down-regulated. Among them, P-value (Differentially expressed) of RRM1 is 0.046, and RRM2 is 0.021, while EG5 (KIF11) is 0.323. Our microarray results have been successfully loaded into a public repository named ‘ArrayExpress’, Data Review URL: http://www.ebi.ac.uk/arrayexpress/experiments/E-MTAB-3495/.

**Fig 1:**
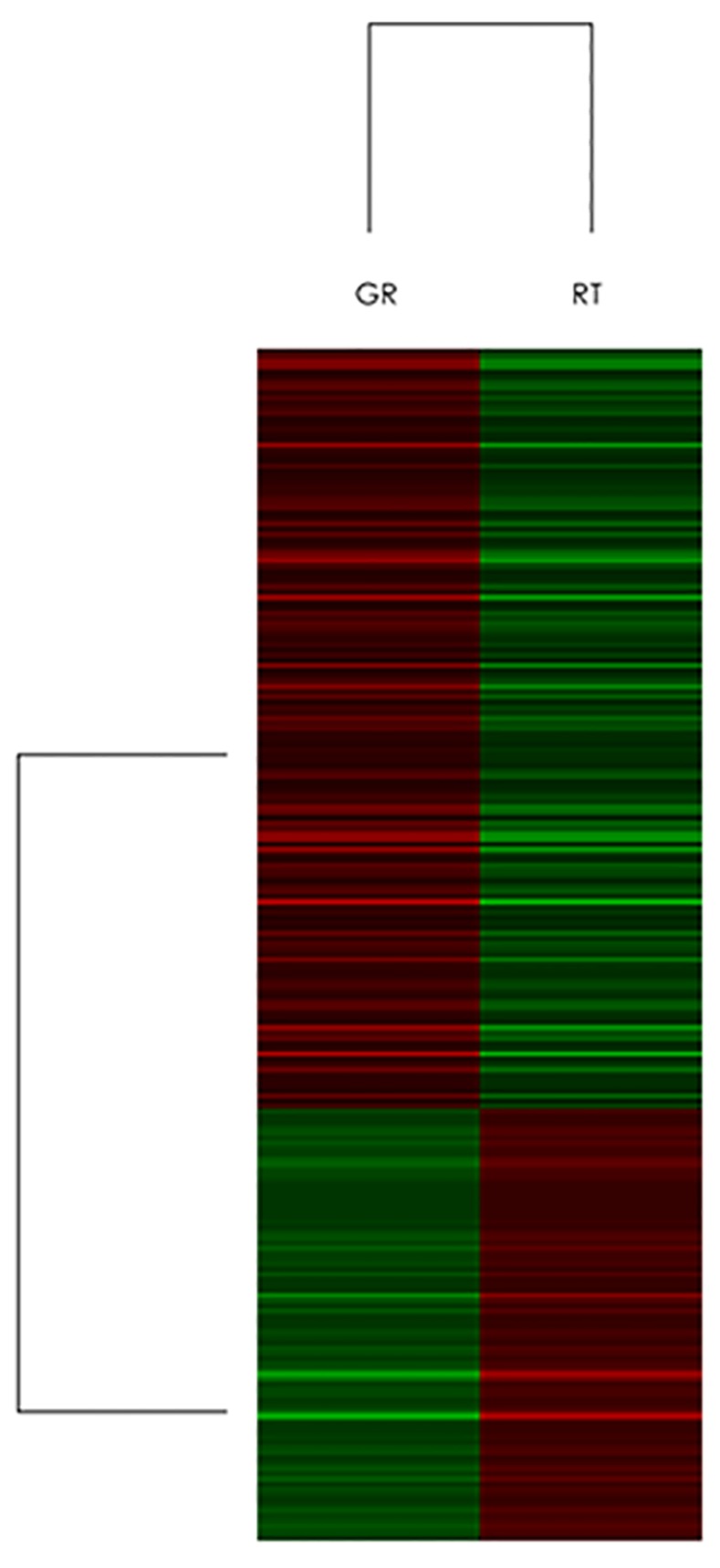
Clustering analysis of microarray. Clustering was performed to visualize the correlations between the replicates and varying sample conditions. Up- and down-regulated genes are represented in red and green colors, respectively. A subset of differential genes was selected for clustering analysis. An intensity filter was used to select genes where the difference between the maximum and minimum intensity values exceeds 1000 among all microarrays. For this microarray project, the number of genes clustered was 227.

Clustering was performed to visualize the correlations among the replicates and varying sample conditions. A subset of differential genes were selected for clustering analysis. An intensity filter was used to select genes where the difference between the maximum and minimum intensity values exceeds 1000 among all microarrays. For this microarray project, the number of genes clustered was 227 (Fig 1).

### RRM1 and RRM2 expression analysis in bladder cancer tissues and cell lines

**Fig 2:**
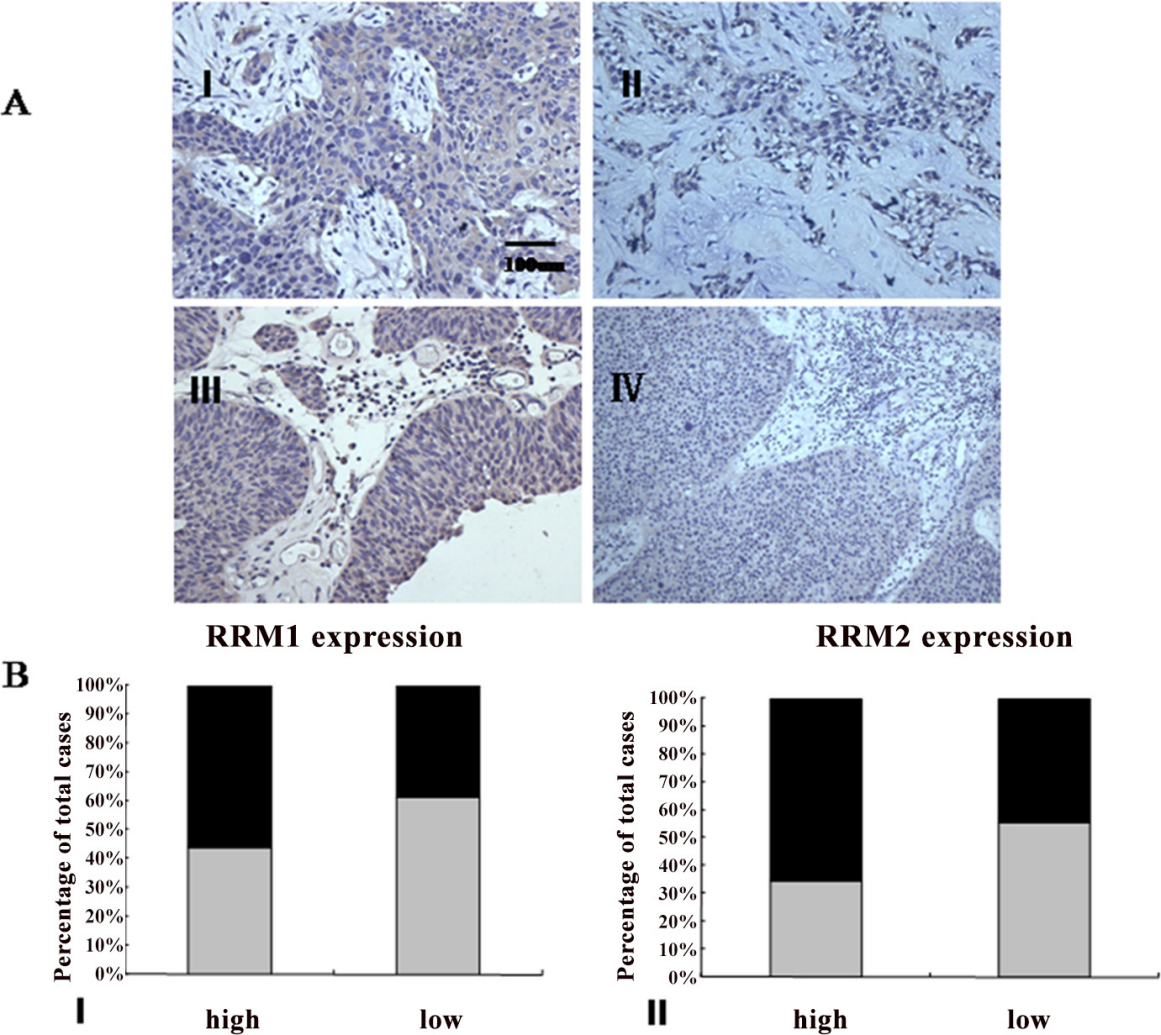
Expression of RRM1 and RRM2 in clinical bladder samples. A:Immunohistochemical staining of RRM1 and RRM2 in clinical tissues. RRM1 and RRM2 immunostaining was considered positive if >10% of the cytoplasm of cancer cells showed weak or greater intensity. (I) High expression of RRM1 in clinical bladder cancer. (II) Low expression of RRM1 in clinical bladder cancer (III) High expression of RRM2 in clinical bladder cancer. (Ⅳ) Low expression of RRM2 in clinical bladder cancer (all figures were captured at 400× magnification). B: (I) Bar graph illustrates combined immunostaining score for RRM1 expression according to tumor grade. (II)Bar graph illustrates combined immunostaining score for RRM2 expression according to tumor stage (The color of black and gray represent weakly and strongly positive, respectively).

**Fig 3:**
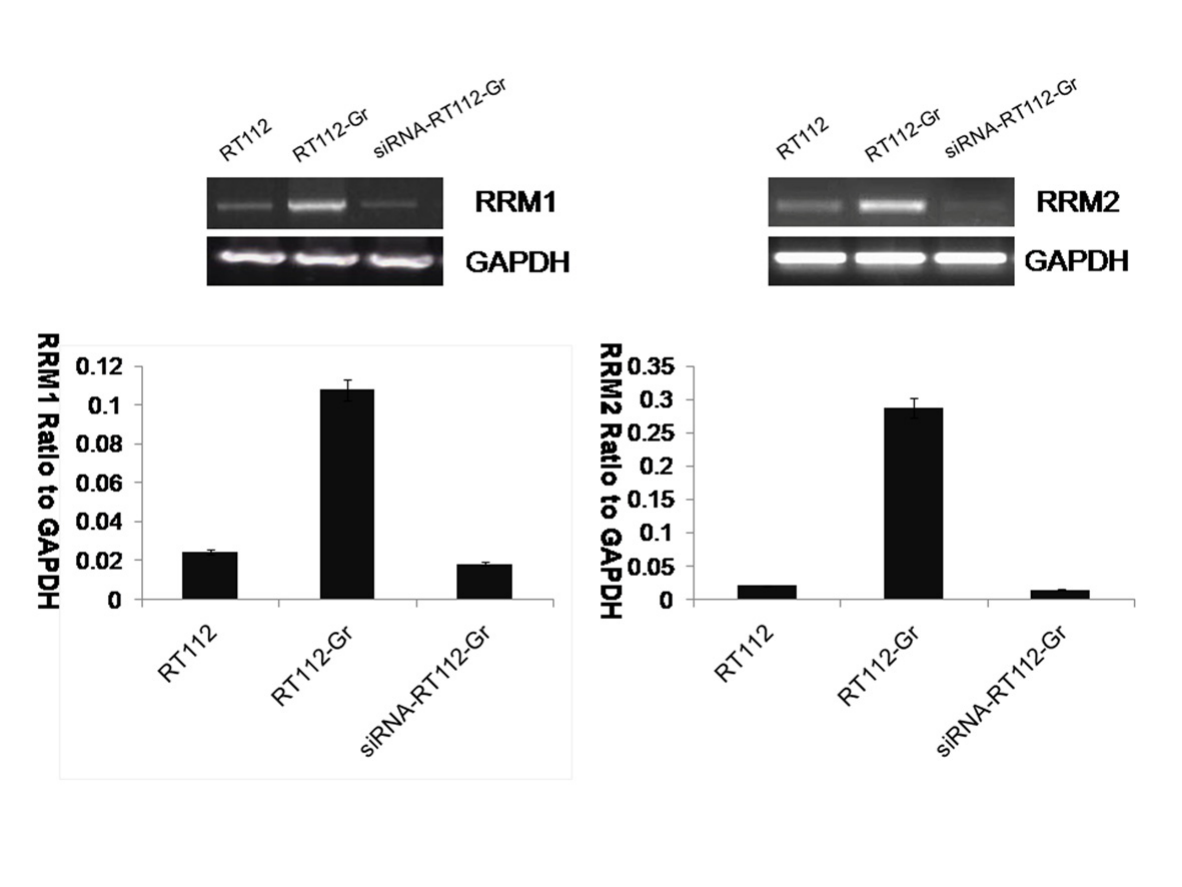
Expression of RRM1 and RRM2 in bladder cancer cell lines. RT-PCR for RRM1 and RRM2 mRNA expression in bladder cancer cell lines. GAPDH served as the loading control. Bar graph illustrates RRM1 and RRM2 mRNA expression in RT112、RT112-Gr and siRNA-RT112-Gr bladder cancer cell lines. (*p* < 0.001).

Immunohistochemical analysis was performed to assay RRM1 and RRM2 expression in surgically resected specimens of bladder cancer. RRM1 and RRM2 staining was localized in the cytoplasm. There were 98 male and 42 female, with 65 low-grade cases and 75 high-grade cases in histological grade. The staining reactivity of anti-RRM1 and anti-RRM2 were showed in the Table 1 and Fig 2, respectively. RT-PCR analysis was plotted to identify RRM1 mRNA and RRM2 mRNA expression in RT112 and RT112-Gr cell lines. RT-PCR further demonstrated that RT112-Gr cells had significant increases in the levels of RRM1 and RRM2 mRNA compared with the parental cells (*p*<0.001), respectively (Fig 3).

**Table 1:** Expression of RRM in relation to clinicopathological characteristics of patients with Bladder Cancer.

Variables		RRM1 staining			RRM2 staining		
	Total	Low expression	High expression	P	Low expression	High expression	P
		No. (%)	No. (%)		No. (%)	No. (%)	
Gender				0.742			0.338
Male	98	46(46.9)	52(53.1)		52 (53.1)	46(46.9)	
Female	42	21(50.0)	21(50.0)		26(61.9)	16(38.1)	
Age				0.675			0.766
<60	59	27(45.8)	32(54.2)		32(54.2)	27(45.8)	
≥60	81	40(49.4)	41(50.6)		46(56.8)	35(43.2)	
Tumor Grade				0.039			0.014
High	75	42(56.0)	33(44.0)		49(65.3)	26(34.7)	
Low	65	25(38.5)	40(61.5)		29(44.6)	36(55.4)	
TNM stage				0.007			0.009
T1/T2	73	27(37.0)	46(63.0)		33(45.2)	40(54.8)	
T3/T4a	67	40(59.7)	27(40.3)		45(58.4)	22(28.6)	

RRM1 and RRM2 staining was localized in the cytoplasm. Tumor grade and stage were evaluated using standard hematoxylin and eosin (H&E) staining.

### siRNA-mediated Knockdown of RRM1/2 in RT112-Gr bladder cancer cells

**Fig 4:**
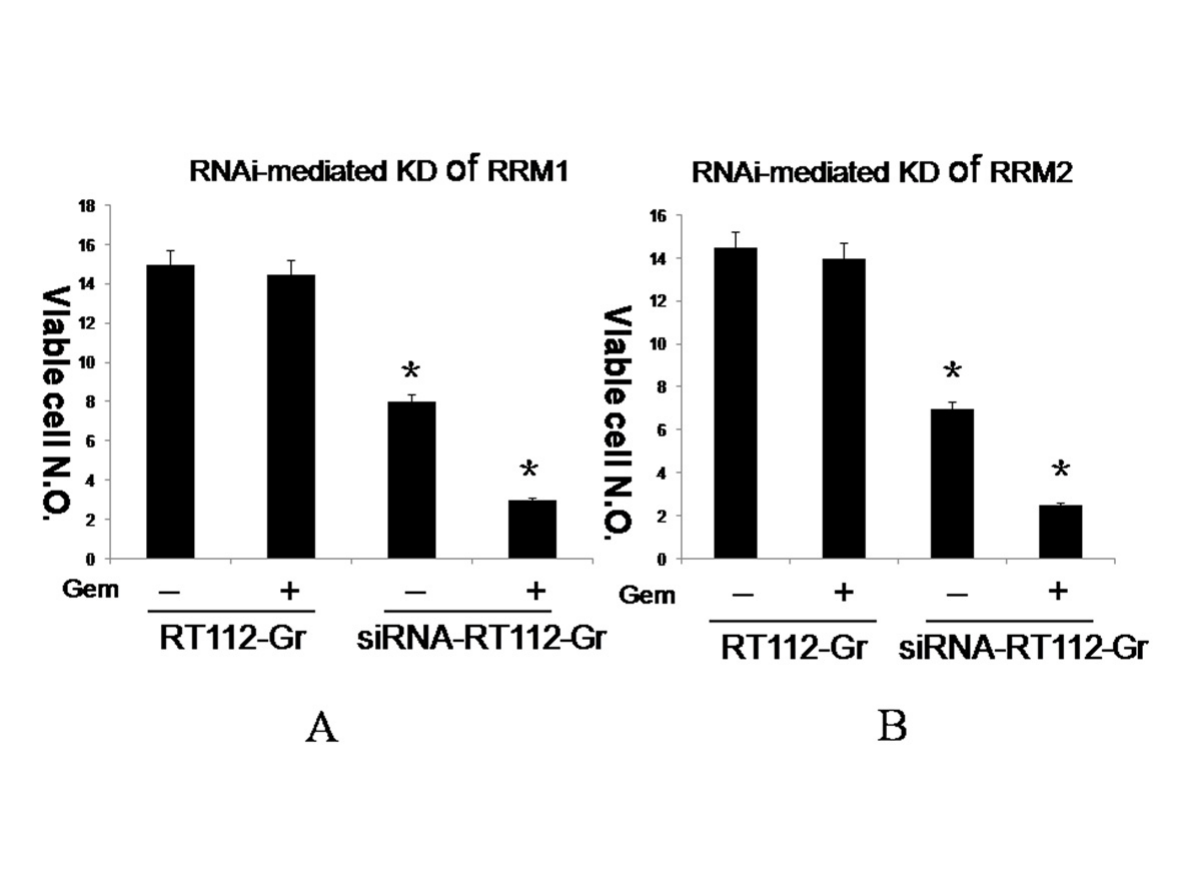
RNAi-mediated Knockdown of RRM1/2 in RT112-Gr bladder cancer cells. RT112-Gr cells were left untreated or were treated with 45nM Gemcitabine (GEM), RNAi-mediated Knockdown of RRM1 (A) and RRM2 (B) in RT112-Gr bladder cancer cells were left untreated or were treated with 45nM Gemcitabine (GEM). Seventy-two hours later viability was analyzed by Trypan blue staining. RNAi-mediated Knockdown of RRM1 (A) and RRM2 (B) +GEM, inhibiting tumor effect is the best among the four groups. Quantification of each value is from triplicate independent experiments. (*p* < 0.01).

To assess the effects of RRM1/2 on RT112-Gr bladder cancer cell lines, RRM1/2 gene were Knockdown, respectively (Fig 3). RT112-Gr cells were left untreated or were treated with 45nM Gemcitabine (GEM), RNAi-mediated Knockdown of RRM1 and RRM2 in RT112-Gr bladder cancer cells were left untreated or were treated with 45nM Gemcitabine (GEM). Seventy-two hours later viability was analyzed by Trypan blue staining. RNAi-mediated Knockdown of RRM1 and RRM2 +GEM, inhibiting tumor effect is the best among the four groups. Quantification of each value is from triplicate independent experiments (Fig 4).

### Antiproliferative effects of S(MeO)TLC and Gemcitabine on Gemcitabine-Resistant bladder cancer cell lines

**Fig 5:**
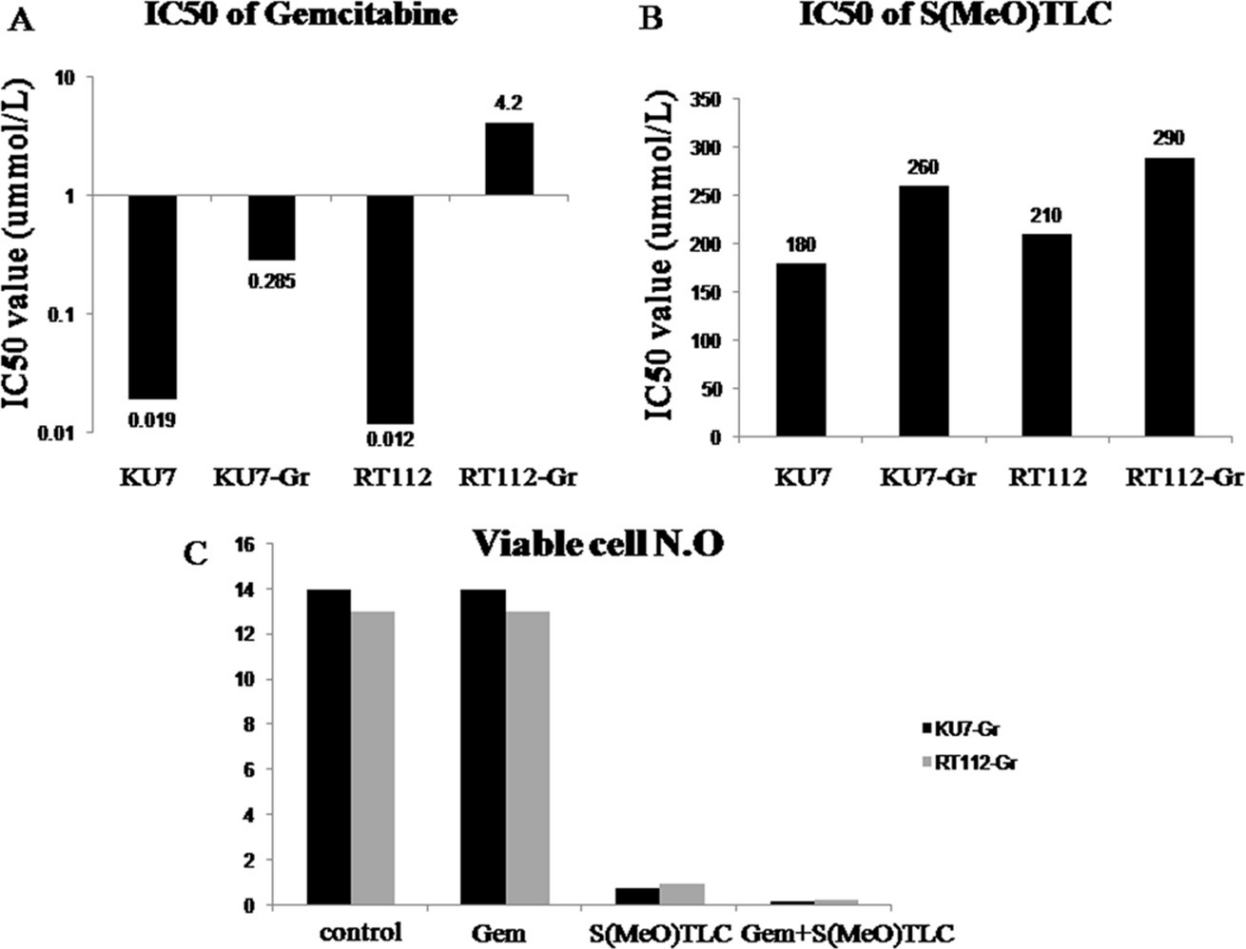
S(MeO)TLC overcomes Gemcitabine resistance. A: The IC50s of Gemcitabine in KU7、KU7-Gr、RT112 and RT112-Gr in 72 hours. B: The IC50s of S(MeO)TLC of KU7、KU7-Gr、RT112 and RT112-Gr in 72 hours. C: KU7-Gr and RT112-Gr cells were left untreated or were treated with 45nM Gemcitabine (GEM), 0.5μM S(MeO)TLC or both together (GEM+ S(MeO)TLC). Seventy-two hours later viability was analyzed by Trypan blue staining. S(MeO)TLC and GEM+ S(MeO)TLC, inhibiting tumor effect is the best among the four group. Quantification of each value is from triplicate independent experiments. (*p* < 0.01).

To assess the anti-proliferative effects of gemcitabine and S(MeO)TLC on bladder cancer cell lines RT112-Gr, they were left untreated or were treated with 45nM gemcitabine, 0.5μM S(MeO)TLC or both together (gemcitabine + S(MeO)TLC) in 24 hours, 48 hours and 72 hours, respectively. Gemcitabine and S(MeO)TLC suppressed cell growth in a time-dependent manner and displayed the most prominent suppression character in the 72 hours. The IC50s of RT112 and RT112-Gr against gemcitabine in 72 hours were 0.012μM and 4.2μM, while IC50s of RT112 and RT112-Gr against S(MeO)TLC were 210μM and 290μM, respectively (Fig 5).

### Induction of apoptosis by S(MeO)TLC and Gemcitabine in Gemcitabine-Resistant bladder cancer cell lines

**Fig 6:**
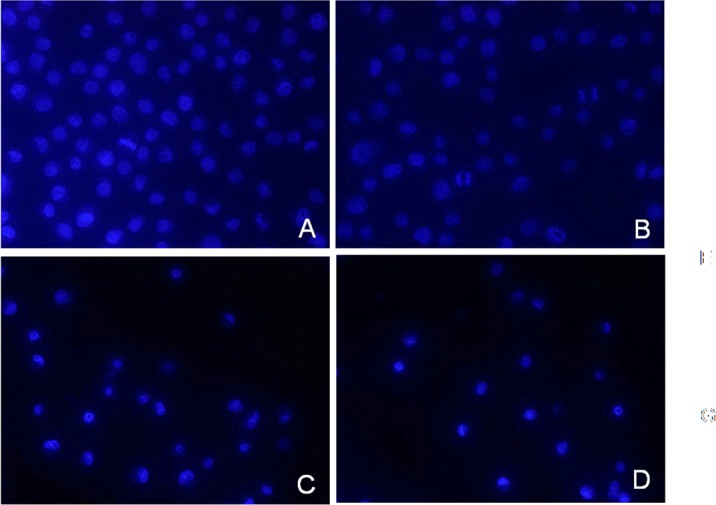
S(MeO)TLC induces Gemcitabine resistance bladder cancer cells apoptosis. A: RT112-Gr cells were left untreated; B: RT112-Gr cells were treated with 45nM Gemcitabine; C: RT112-Gr cells were treated with 0.5μM S(MeO)TLC; D: RT112-Gr cells were treated with both together (45nM Gemcitabine + 0.5μM S(MeO)TLC) for the indicated times and then nuclear morphology was examined with Hoechst staining and visualized by fluorescent microscopy.

Hoechst 33342 nuclear staining demonstrated that typical monopolar spindle mitotic cells with a characteristic rosette-like phenotype were observed in the mitotic phase at 24 hours after exposure to 45nM gemcitabine, 0.5μM S(MeO)TLC, 45nM gemcitabine+0.5μM S(MeO)TLC or vehicle and distinctive apoptotic cells with nuclear condensation and fragmentation were also identified at 48 h after exposure (Fig 6).

Trypan blue staining confirmed that in gemcitabine + S(MeO)TLC group and S(MeO)TLC group cell viability were significantly decreased in RT112-Gr and KU7-Gr cells as compared with the control group (Fig 5).

### Effects of S(MeO)TLC and Gemcitabine in the bladder cancer xenograft model

**Fig 7:**
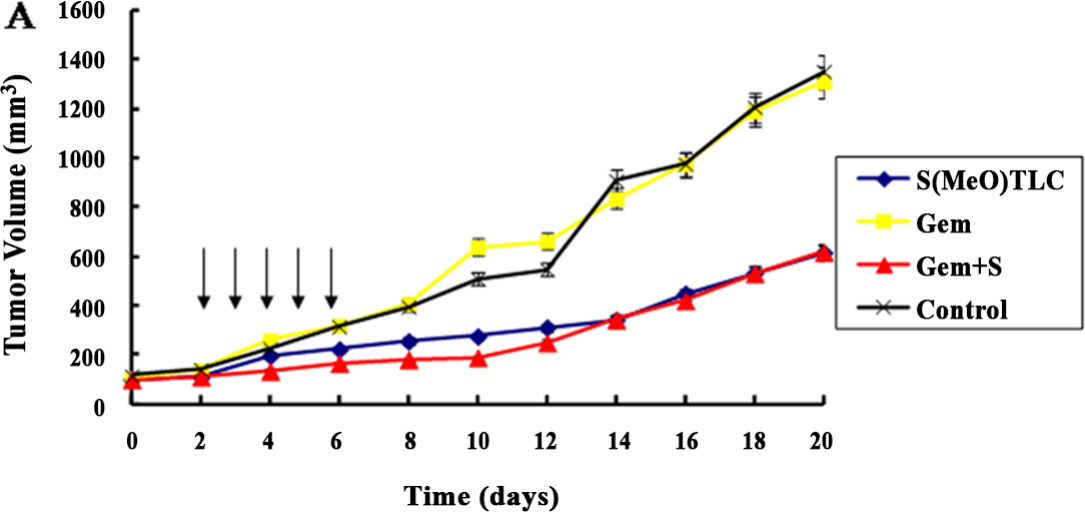
Anticancer activity of S(MeO)TLC in subcutaneous xenograft tumors. A. After successful establishment of subcutaneous xenograft tumors, DMSO (vehicle control), 20 mg/kg S(MeO)TLC, 50 mg/kg Gemcitabine or both(S(MeO)TLC + GEM) were administered intraperitoneally daily for 5 days (arrows). Tumor volumes were measured every other day. B. The mean body weights of mice were assessed every other day. There is no significant difference among three groups (*P*>0.05).

For the initial analysis of the anticancer activity of S(MeO)TLC in subcutaneous xenograft models, tumor volume were evaluated after treatment with DMSO (vehicle control), 20 mg/kg S(MeO)TLC, 50 mg/kg gemcitabine or 20 mg/kg S(MeO)TLC +50mg/kg gemcitabine. After 3 weeks of observation it was found that S(MeO)TLC(20mg/kg) +gemcitabine(50mg/kg) and S(MeO)TLC (20mg/kg) groups prominently suppressed tumor growth in comparison with gemcitabine(50mg/kg) and DMSO (vehicle control). Body weight loss was not observed in any of the treatment groups. Necrosis was observed on the tumor surface of S(MeO)TLC (20 mg/kg) and S(MeO)TLC(20mg/kg) +gemcitabine(50mg/kg) treatment group, although no obvious necrosis was observed in the control group. Tumor volume changes of the mice were compared between treatment and control groups, the difference in the mean final tumor volume among the four groups was statistically significant (Fig 7).

## Discussion

Chemoresistance is a major cause of failure in treatment for bladder cancer with gemcitabine. Therefore, it is extremely important to clarify the mechanism of chemoresistance and identify predictive markers of inherent and acquired chemoresistance to gemcitabine [24]. Ribonucleotide reductase (RR) is a heterotetramer, and activity requires two 90kDa large subunits and two 45kDa small subunits. The enzyme is crucial for the supply of deoxynucleoside for de novo synthesis of DNA and so for cell proliferation. It consists of the dimerised large and small subunits, M1 and M2. RRM1 is a large peptide chain(α), while RRM2 is a small protein subunits of RR (β). Although the enzymatic activity of RR is modulated by levels of RRM2, RRM1 could play a key role among the 2 subunits in the course of gemcitabine treatment[9, 13]. The role of RRM1 in RR formation has also made it an attractive molecular target for the development of chemotherapeutic agents, such as gemcitabine[13].

Gene expression profiling by microarray analysis provides a powerful means of obtaining an overview of gene expression and physiological processes involved in responses to particular stimuli. In the current study, we established gemcitabine-resistant human bladder cancer cell lines RT112-Gr and KU7-Gr, RT112-Gr cells were 350-fold less sensitive to gemcitabine than the parental cell lines, while KU7-Gr cells were 15-fold less sensitive to gemcitabine than the parental cell lines. So we choose RT112-Gr cells as a subject in the following experiments. microarray analysis was used to examine gene expression changes between RT112 and RT112-Gr cells. Our results indicate that 442 genes, including RRM1 and RRM2, were upregulated, while 235 genes downregulated in RT112-Gr cells. Eg5 change was not obvious. Our present study indicates that RRM1 and RRM2 are involved in gemcitabine resistance in human bladder cancer. In the cell, gemcitabine is phosphorylated to monophosphate, diphosphate, or triphosphate before its incorporation into DNA, which is necessary for its growth inhibiting activity. The diphosphorylated form of gemcitabine acts as a RR inhibitor, which is the cause of gemcitabine cytotoxic activity. RR increases the deoxynucleoside triphosphate (dNTP) pool in the cells, which could lead to decreased incorporation of dNTP analogues such as triphosphorylated gemcitabine into DNA and might reduce the antitumor effect of gemcitabine[9]. A number of preclinical and clinical studies have demonstrated that RRM1 could be the targeted molecule to regulate gemcitabine resistance. Furthermore, its expression levels could be a useful indicator of gemcitabine resistance. Shin Nakahira et al reported that increased RRM1 expression was significantly associated with antitumor effects and with poor survival after treatment with gemcitabine in pancreatic cancer patients[9]. Hao Xie et al reported that after surgical resection in pancreatic adenocarcinoma, to achieve the best survival, the level of RRM1 expression may be used to stratify patients to receive either adjuvant gemcitabine or non-gemcitabine adjuvant therapy. This is consistent with the results from early stage NSCLC studies[13], and this implication is supported by the role of RRM1 as a tumor suppressor[12]. Moreover, RRM2 is known to be involved in chemoresistance. Suppression of RRM2 could sensitize colon and pancreatic cancer cells to chemotherapeutic agents[25]. The relationship between RRM2 expression and chemotherapeutic effect in clinical samples has also been investigated. Itoi et al examined RRM2 mRNA levels in biopsy specimens from 35 patients with unresectable pancreatic cancer in a prospective study, and found that the response rate to gemcitabine chemotherapy was significantly higher in patients with low RRM2 expression[26]. However, none of the previous studies have investigated the expression of RRM in bladder cancer.

Our study found that RRM1 and RRM2 were expressed in clinical bladder cancer tissues and bladder cancer cell lines. It is indicated that the expression of RRM1 and RRM2 was significantly higher in low-grade tumors than high-grade tumors. Our data suggested that RRM1 and RRM2 overexpression could be associated with the progression of bladder cancer. In bladder cancer cell lines, RT-PCR results demonstrated that RT112-Gr cells had significantly increased in the levels of RRM1 and RRM2 mRNA compared with the parental cells(*p*<0.001), respectively. To assess the effects of RRM1/2 on RT112-Gr bladder cancer cell lines, RRM1/2 gene were knockdown. Our study indicated that after knockdown of RRM1/2, RT112-Gr bladder cancer cell lines sensitive to gemcitabine again, indicating the overexpression of RRM was associated with gemcitabine resistance, and might be a novel candidate biomarker for gemcitabine resistance. Data from numerous clinical studies showed that RRM1/2 expression in tumor cells is inversely correlated to the sensitivity of the tumor cells to gemcitabine therapy [9, 11, 12]. For example, Woon-Gye Chung et al reported that the increased RRM1 expression was likely responsible for the resistance to gemcitabine in the TC-1-GR(gemcitabine resistance TC-1) cells, which was further supported by the observation that transfection of RRM1 siRNA into TC-1-GR cells made the cells more sensitive to gemcitabine HCl [27]. Zhang M et al reported that Small interfering RNA-mediated RRM2 knockdown significantly reversed SKOV3/DDP cell resistance to cisplatin [28].

Evidence showed that patients with gemcitabine resistance would face a worse outcome. Therefore, the treatment of gemcitabine resistance bladder cancer is very important. Increasing studies demonstrated a better result using targeting therapy in treating bladder cancer. Our early study found Eg5 inhibitors as targeted drugs in vivo and in vitro treatment of bladder cancer can have good curative effect, furthermore, we initially demonstrated the anticancer effect of Eg5 inhibitor on gemcitabine resistance bladder cancer in the present paper.

Eg5 is a homotetramer motor formed by the antiparallel arrangement of two dimers. The Eg5 tetramer has the ability to crosslink antiparallel microtubules emanating from the two centrosomes at G2/M[29]. The primary function of Eg5 is to form the bipolar spindle during early prometaphase; failure to separate the duplicated centrosomes leads to mitotic arrest and ultimately triggers apoptotic cell death in certain tumor cell lines[30, 31]. Inhibition of Eg5 blocks centrosome separation and thereby inhibits cell division[32]. Eg5 inhibitors are expected to be potent anticancer drugs with fewer side effects than conventional mitotic inhibitors that directly inhibit polymerization of microtubules[23,33,34]. A number of small molecule inhibitors of Eg5 have been developed. Recently, it has been found by ourselves and other investigators that S-(methoxytrityl)-L-cysteine (S(MeO)TLC), a derivative of S-trityl-L-cysteine (STLC), can specifically inhibit Eg5, as does STLC, and induce monopolar mitotic spindle formation, but it is 10 times more potent than STLC[14, 15].

Previous reports cited that Eg5 inhibitors can exhibit powerful anticancer activity in taxol-resistant cancer cells, and they can overcome taxane resistance[15]. Good JA et al recently reported that Eg5 inhibitors can induced complete tumor regression in nude mice explanted with lung cancer patient xenografts[35]. Marcus et al previously reported that docetaxel-resistant PC3 cells remained sensitive to the effects of Eg5 inhibition by the use of an antisense oligonucleotide[36]. It has been also reported that Eg5 is a potential therapeutic target in prostate cancer and Eg5 inhibitor(STLC) remains effective in docetaxel-resistant prostate cancer cells[19]. None has been reported about the antitumor effects of S(MeO)TLC on gemcitabine resistance bladder cancer.

In the present study, our data demonstrated that S(MeO)TLC exhibited powerful antitumor effects on RT112-Gr cell lines. The most surprising finding of our study is that in RT112-Gr cell lines after the application of S(MeO)TLC alone, the cancer inhibiting effect is similar with the S(MeO)TLC plus gemcitabine group. This finding has never been mentioned in previous reports. The mechanism is unclear. Our results suggest the potential efficacy of S(MeO)TLC for clinical therapy against bladder cancer, which is a promising anticancer strategy.

Since S(MeO)TLC exhibited excellent therapeutic potency in bladder cancer cell lines, we determined its inhibitory activity in subcutaneous xenograft tumor models. In our previous reports, histopathological analysis revealed that treatment with S(MeO)TLC led to tumor growth inhibition by inducing mitotic arrest with monopolar spindle phenotypes[14]. Our data indicates that S(MeO)TLC has a strong anticancer action in subcutaneous xenograft tumor models. It is especially interesting that the anticancer effect of S(MeO)TLC alone and S(MeO)TLC plus gemcitabine are stronger than other groups’ in vivo models. We were surprised to find that S(MeO)TLC plus gemcitabine and S(MeO)TLC group induced obvious necrosis in subcutaneous xenograft tumors. Furthermore, an intraperitoneal dose of 20 mg/kg S(MeO)TLC and 50mg/kg gemcitabine are proved to be safe without any adverse events or weight loss. The reason why Eg5 inhibitor exhibited significant anticancer efficacy in gemcitabine resistant bladder cancer cell lines may be because Eg5 and RRM exist gene coexpression in bladder cancer, and we will study the relationship in the following research.

Viewed in toto, Eg5 could be a good target for bladder cancer chemotherapy. Our current study confirmed that S(MeO)TLC, as a novel Eg5 inhibitor, exhibited significant anticancer efficacy in gemcitabine resistant bladder cancer cell lines both in vitro and in vivo. Furthermore, it is especially interesting that the inhibiting tumor effect of S(MeO)TLC plus gemcitabine is similar as S(MeO)TLC alone in vitro and in vivo models. On the basis of our findings we believe that S(MeO)TLC is a promising novel anticancer agent for the treatment of gemcitabine resistant bladder cancer.

## Conclusion

This is the first report of RRM1 and RRM2 protein overexpression in bladder cancer. RRM1 and RRM2 gene expression might be a predictive marker for the efficacy of gemcitabine therapy in bladder cancer patients. S(MeO)TLC, as a novel Eg5 inhibitor, exhibited significant anticancer efficacy in gemcitabine resistant bladder cancer cell lines both in vitro and in vivo, and may provide a new therapeutic option to overcome chemoresistance in bladder cancer.
